# Blind Image Blur Assessment Using Singular Value Similarity and Blur Comparisons

**DOI:** 10.1371/journal.pone.0108073

**Published:** 2014-09-23

**Authors:** Qing-Bing Sang, Xiao-Jun Wu, Chao-Feng Li, Yin Lu

**Affiliations:** 1 Key Laboratory of Advanced Process Control for Light Industry (Ministry of Education), School of Internet of Things Engineering, Jiangnan University, Wuxi, Jiangsu, China; 2 Computer Science Department, Texas Tech University, Lubbock, Texas, United States of America; Institute of Psychology, Chinese Academy of Sciences, China

## Abstract

The increasing number of demanding consumer image applications has led to increased interest in no-reference objective image quality assessment (IQA) algorithms. In this paper, we propose a new blind blur index for still images based on singular value similarity. The algorithm consists of three steps. First, a re-blurred image is produced by applying a Gaussian blur to the test image. Second, a singular value decomposition is performed on the test image and re-blurred image. Finally, an image blur index is constructed based on singular value similarity. The experimental results obtained on four simulated databases to demonstrate that the proposed algorithm has high correlation with human judgment when assessing blur or noise distortion of images.

## Introduction

Digital images produced by digital cameras are widely used. The objective of image processing in many applications is the search for an in-focus, noise-free version of an unknown original. It is necessary to assess the quality of images if qualified images are expected to be sorted out and if unqualified ones are dumped automatically. The naked eye is undoubtedly a good classifier, but an objective method saves time and should simplify the task. Objective IQA methods fall into three categories: Full Reference (FR), Reduced Reference (RR) and No Reference (NR) or Blind Reference (BR). FR and RR need all of, or at least part of, the information regarding the reference image. However, in many application occasions, the information regarding the reference image is inaccessible. Hence, the NR method is comparatively more valuable.

In recent years, research into no-reference image blur assessment methods has been notably active. A variety of no-reference blur indexes have been proposed in the literature. For example, in [1], an image sharpness index is proposed that is based on the notion of just noticeable blur (JNB). Rania Hassen et al in [2] propose a new sharpness measure by utilizing local phase coherence (LPC) evaluated in the complex wavelet transform domain. In [3], the authors present a no-reference image blur metric, which utilizes a probabilistic model to estimate the probability of detecting blur at the edges of an image; the information is later pooled by computing the cumulative probability of blur detection (CPBD).

The singular value decomposition (SVD) method has been successfully applied to FR IQA. The existing FR methods based on SVD are primarily divided into two categories. One category uses the singular value to assess image quality. For example, the MSVD algorithm proposed in [4] uses the amount of change of the singular value as the image quality evaluation criteria. The other category uses the left singular vectors and right singular vectors to assess image quality [5]. This paper analyses the relationship between the change of the singular value and the blurred degree, and a new method for image blur index is suggested based on a singular value similarity that does not require the reference image.

## Singular Value Similarity

Every grayscale image can be considered as a matrix. Any m×n real matrix *A* can be decomposed into a product of three matrices, i.e., 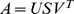, where *U* and *V* are orthogonal matrices, 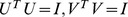, and 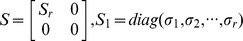, where *r* is the rank of *A*. The diagonal entries of *S_r_* are known as the singular values of *A*, the columns of *U* are called the left singular vectors of *A*, and the columns of *V* are called the right singular vectors of *A*. This decomposition is known as the Singular Value Decomposition (SVD) of *A*. The SVD is one of the most useful tools of linear algebra with several applications for image processing, including image de-noising, compression, and watermarking.

In this paper, We imitate the SSIM [8] algorithm and use the similarity of the singular value vectors between source image and the distorted image to represent the quality of distorted image, and define a novel *NSVD* index for IQA as following:
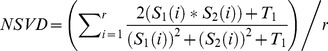
(1)where 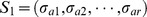 and 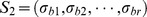 are the singular value vectors of source Image 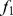 and the distorted image 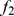, and *r* is the number of the singular value. 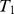 is a small positive constant to increase the stability of *NSVD* (such a consideration was also included in SSIM [8]). Formula (1) is a commonly used measure to define the similarity of two positive real numbers [8] and its result ranges within (0, 1].

**Figure 1:**
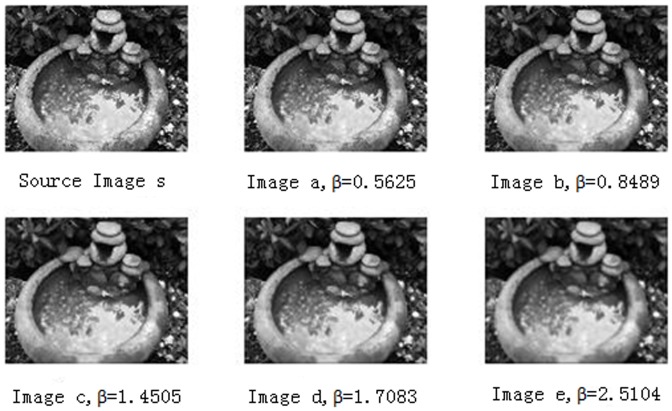
Source Image s and its different degrees of blurring distorted images.

To explain our propsed *NSVD* index, we arbitrarily selected a source image and five blurred versions of it from the LIVE2 database [7], as shown in Figure 1, wherethe degree of blur is sorted in ascending order from Image a to Image e. Figure 1 consists of blurred images with R, G, and B components that were filtered using a circularly symmetric 2-D Gaussian kernel of standard deviation*β*. The values of *β* are given in Figure 1. The greater the value of standard deviation *β*, the greater the blurred degree.

**Figure 2:**
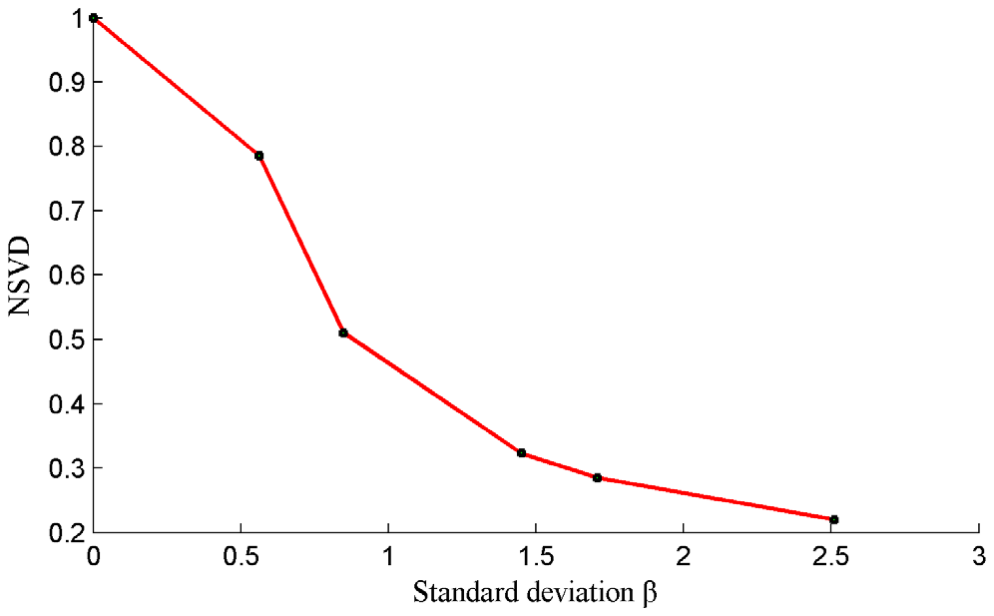
Plot of standard deviationβ versus NSVD of **Figure 1**.

We calculate the values of the *NSVD* of Figure 1 using the formula (1) given in Table 1, and the values of standard deviation*β*are also listed in Table 1. A scatter plot of *β* versus *NSVD* are given in the Figure 2. The greater the value of standard deviation *β*, the greater the blurred degree. From Figure2, it can be observed that *β* and *NSVD* have an approximate inverse proportion relationship, and *NSVD* can be used as an FR IQA index to measure blurred degree of images.

**Table 1:** The values of *β*, NSVD of Figure 1.

image	s	a	b	c	d	e
*β*	0	0.5625	0.8489	1.4505	1.7083	2.5104
*NSVD*	1	0.9499	0.7798	0.5139	0.2881	0.1527

## Gaussian Blur

A Gaussian blur (also known as Gaussian smoothing) has the effect of reducing an image's high-frequency components and is widely used to reduce the detail of an image to produce a blurred image or a de-noising image.

**Figure 3:**
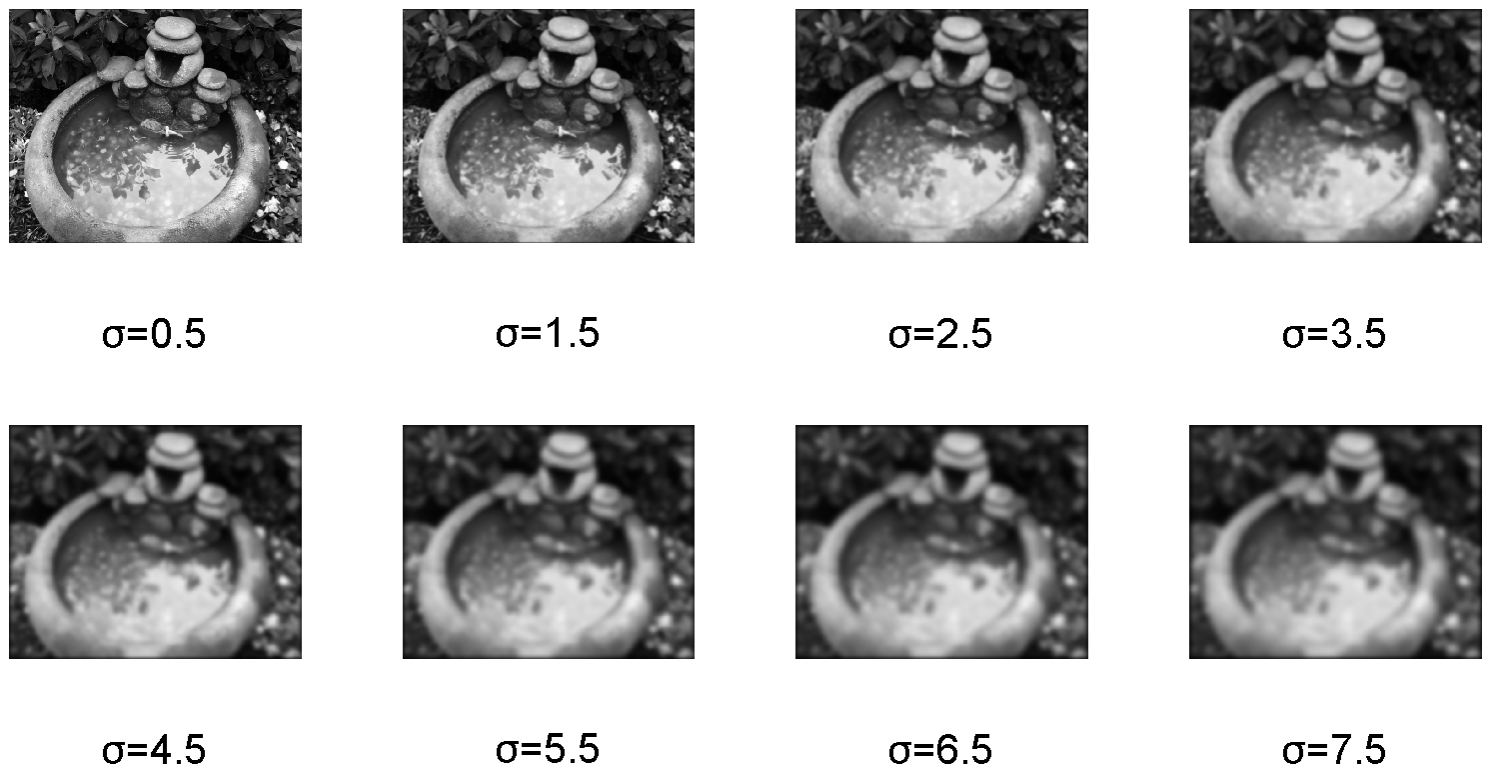
Re-blurred images using different values of σ.

**Figure 4:**
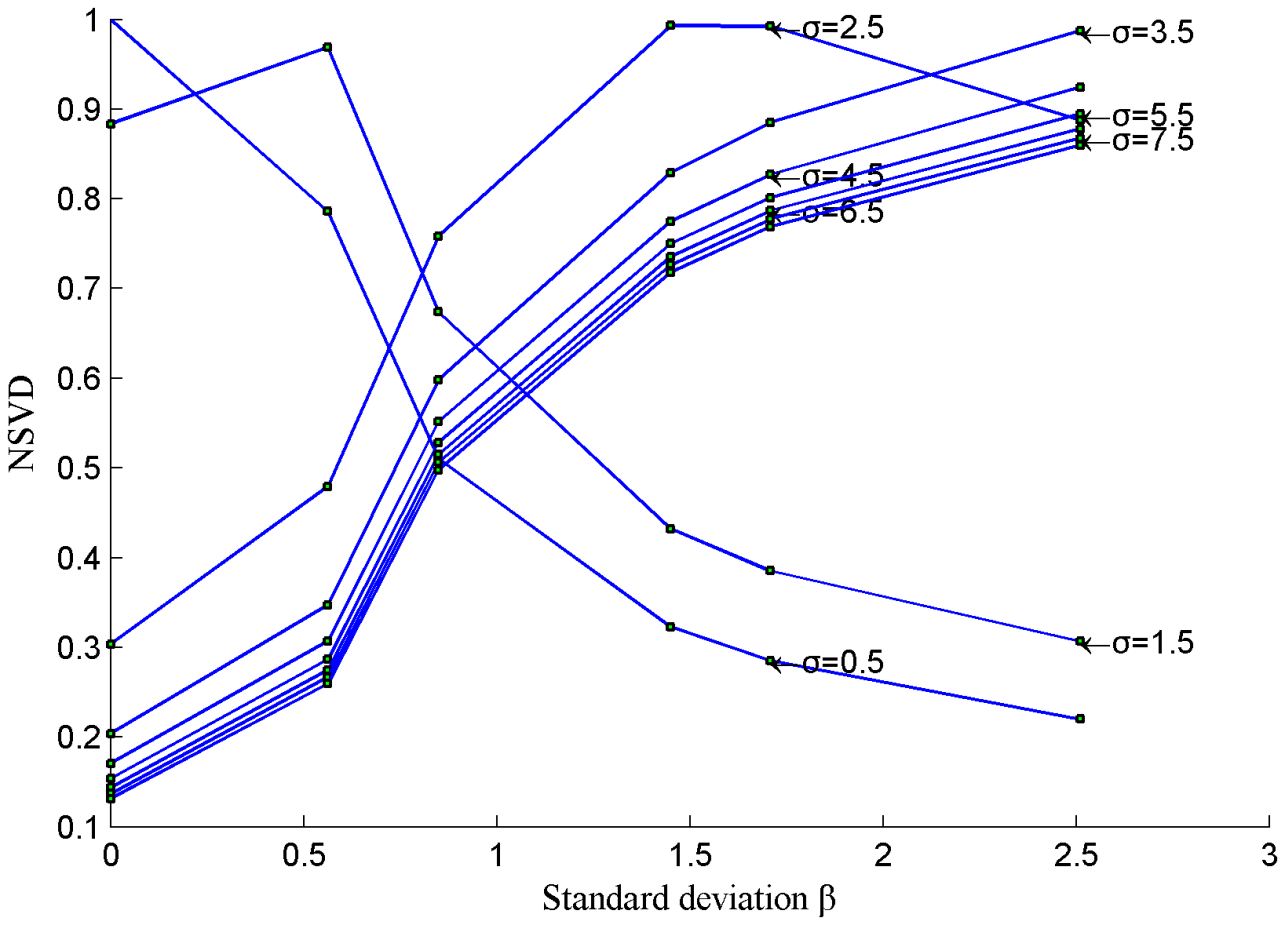
Plots of standard deviationβ versus NSVD of **Figure 1**, when the re-blurred images in **Figure 3** are regarded as a ‘reference image’.

In this paper, we consider to use the difference between blurred image and reblurred image to represent image quality, so the NSVD index between blurred image and reblurred image is used. Several re-blurred images are produced with different standard deviations σ by using the Gaussian blur function in Matlab, as shown in Figure 3. We regard each of these re-blurred images as a ‘reference image’ (similar to ‘Source Image s’ in Figure 1), and calculate the values of *NSVD* for the blurred images in Figure 1. The plots of *β* versus *NSVD* are be shown as Figure 4. It can be observed that when the values of *σ* are greater than 3.5, *β* and *NSVD* have an approximate direct proportion relationship, and NSVD between blurred image and reblurred image reflect the blurred degree of image.

## Constructing the No-Reference Blur Inex

**Figure 5:**
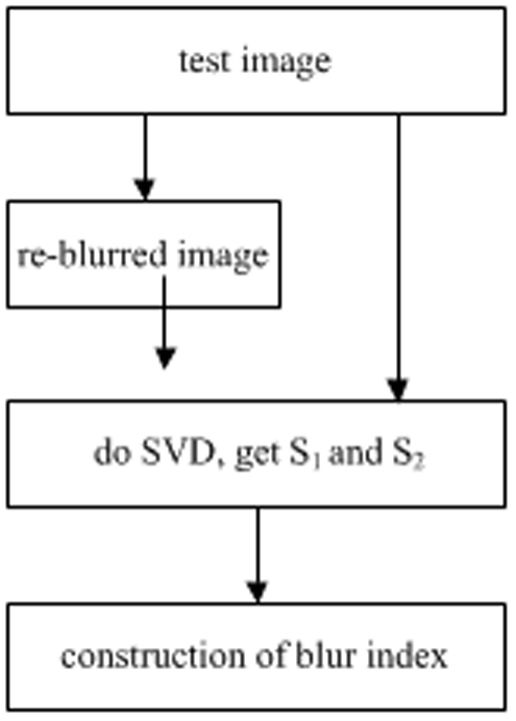
Flowchart of our proposed algorithm.

Figure 5 exhibits a flowchart of the proposed no-reference blur assessment algorithm, and the steps are as follows.

1. First, a re-blurred image is produced by applying a Gaussian blur to a test image with a window size of 11×11 and a standard deviation *σ* set to 5.
2. Singular vectors *S_1_* and *S_2_* are gained by applying Singular Value Decompositions to the test image and re-blurred image.
3. A blurred image quality index is calculated using the formula (1).

## Experimental Results and Analysis

### A. Databases and Metrics for Comparison

Performance of the proposed blur index was evaluated on four blur image databases (CSIQ [6], LIVE2 [7], TID2008 [9], and IVC [10]). The characteristics of these four databases are summarized in Table 2.

**Table 2:** Benchmark test databases for IQA.

Database	Source images	Types	Blurred images	Observers
LIVE2	29	color	145	161
TID2008	25	color	100	838
CSIQ	30	color	150	35
IVC	10	color	20	15

Two commonly used performance metrics were employed to evaluate the competing IQA metrics. The first is the Spearman rank-order correlation coefficient (SROCC), which measures the prediction monotonicity of an IQA metric. This metric operates on the ranked data points and ignores the relative distances between data points. The second metric is the Pearson linear correlation coefficient (CC) between MOS and the objective scores after nonlinear regression. For the nonlinear regression, we used the following mapping function:
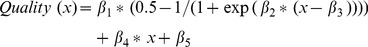
(2)where *x* is the score obtained from the objective metric, and *β_k_* with k = 1,2,3,4,5 are parameters. The fitting, i.e., determination of parameters in [11], is done by the nonlinear regression over dataset.

### B. Tests on the LIVE2 Database

According to the analysis in section 2, formula (1) can be used as an FR IQA method. We tested the formula on the LIVE2 database, which consists of 29 different reference images and 779 distorted images from five distortion categories—JPEG2000 (JP2K), JPEG (JPEG), White Noise (WN), Gaussian Blur (Blur) and Fast fading noise (FF)—along with the associated DMOS, which represent human judgments of image quality; and we then made a comparison using two classical FR IQA metrics, SSIM [8] and PSNR. From Table 3, it can be seen that the index *NSVD* correlate well with human DMOS. On the Blur, JP2K, and JPEG distortion categories, the *NSVD* index delivers a better performance than SSIM and PSNR. However, in the FF and WN categories, its results are somewhat inferior to those of SSIM.

**Table 3:** Performance of NSVD on the LIVE2 database.

Measure	Distortion	NSVD	SSIM	PSNR
SROCC	WN	0.9379	**0.9635**	0.9410
	JPEG	**0.9478**	0.9466	0.8831
	JP2K	**0.9450**	0.9389	0.8646
	Blur	**0.9559**	0.9046	0.7515
	FF	0.8783	**0.9393**	0.8736
CC	WN	0.9490	**0.9824**	0.9173
	JPEG	**0.9472**	0.9462	0.9029
	JP2K	**0.9424**	0.9405	0.8762
	Blur	**0.9449**	0.9004	0.7801
	FF	0.8751	**0.9514**	0.8795

### C. Test on Four Databases

We compared our blur index with three current top no-reference blur indices [1]–[3] on the four simulated blur databases. The experimental results are shown in Table 4, which demonstrates the advantage of our proposed algorithm. Furthermore, our proposed index has the best performance on distortion types of noise compared to the other blur indices.

**Table 4:** Performance comparisons of no-reference blur image quality assessment models on LIVE2, TID2008, CSIQ and IVC databases.

Distortion Type	Measure	Model	LIVE2	TID2008	CSIQ	IVC
Blur	SROCC	JNB [1]	0.8368	0.7045	0.7625	0.7722
		LPC [2]	0.9368	0.8030	0.8931	**0.9022**
		CPBD [3]	0.9437	0.8406	0.8790	0.8404
		**NSVD**	**0.9509**	**0.8969**	**0.9247**	0.8547
	CC	JNB [1]	0.8390	0.7171	0.8572	0.7992
		LPC [2]	0.9239	0.8113	0.8856	**0.9718**
		CPBD [3]	0.9107	0.8316	0.8743	0.8865
		**NSVD**	**0.9537**	**0.9312**	**0.9460**	0.8859
Noise	SROCC	JNB [1]	0.6004	0.2985	0.6077	-
		LPC [2]	0.8147	0.1408	0.2049	-
		CPBD [3]	0.9317	0.4156	0.6523	-
		**NSVD**	**0.9637**	**0.7574**	**0.8841**	-
	CC	JNB [1]	0.6484	0.3355	0.5951	-
		LPC [2]	0.8581	0.1496	0.2238	-
		CPBD [3]	0.9529	0.4104	0.6526	-
		**NSVD**	**0.9706**	**0.8050**	**0.8898**	-

**Figure 6:**
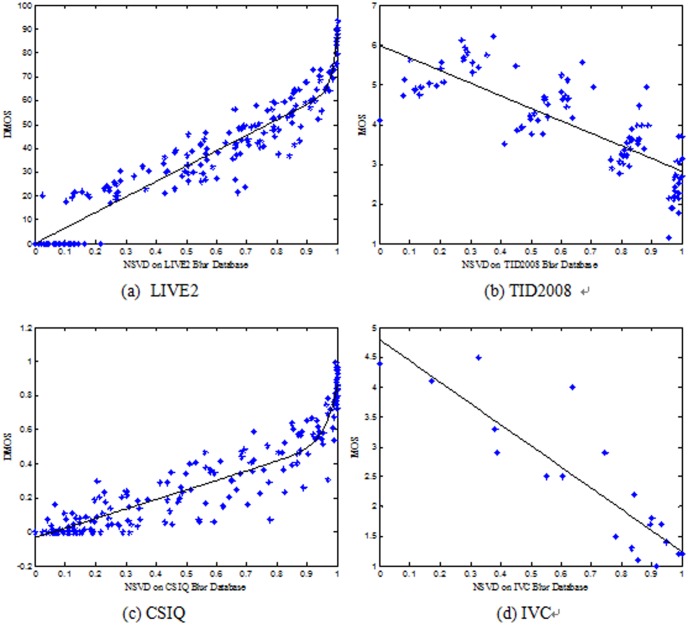
NSVD versus DMOS (MOS) on four blur databases. Each data point represents one test image.

Figures 6 (a)-(d) display the scatter plots of the NSVD objective scores versus DMOS (MOS) on the four blur databases. Each data point represents one test image. The curves shown in Figure 6 were obtained by a nonlinear fitting according to [11]. From Figure 6, one can see that the NSVD index is consistent with human subjective judgments of quality in these distortions.

## Conclusions

In this work, we presented a novel no-reference image blur index based on the singular value similarity. The efficiency of the new algorithm is validated on four simulated blur databases. This algorithm can accurately assess images that have blurring or noise. In addition, the proposed index *NSVD* can also be observed as an FR IQA index.
